# Modifying the minimum criteria for diagnosing amnestic MCI to improve prediction of brain atrophy and progression to Alzheimer’s disease

**DOI:** 10.1007/s11682-018-0019-6

**Published:** 2018-12-04

**Authors:** Eero Vuoksimaa, Linda K. McEvoy, Dominic Holland, Carol E. Franz, William S. Kremen, for the Alzheimer’s Disease Neuroimaging Initiative

**Affiliations:** 1 Institute for Molecular Medicine Finland (FIMM), University of Helsinki, P.O. Box 20 (Tukholmankatu 8), 00014 Helsinki, Finland; 2 Department of Radiology, University of California, San Diego, La Jolla, CA USA; 3 Department of Neurosciences, University of California, San Diego, La Jolla, CA USA; 4 Department of Psychiatry, University of California, San Diego, La Jolla, CA USA; 5 Center for Behavior Genetics of Aging, University of California, San Diego, CA USA; 6 Center of Excellence for Stress and Mental Health, VA San Diego Healthcare System, San Diego, CA USA

**Keywords:** Alzheimer’s disease, Biomarkers, Early detection, Mild cognitive impairment, Neuropsychological testing

## Abstract

Mild cognitive impairment (MCI) is a heterogeneous condition with variable outcomes. Improving diagnosis to increase the likelihood that MCI reliably reflects prodromal Alzheimer’s Disease (AD) would be of great benefit for clinical practice and intervention trials. In 230 cognitively normal (CN) and 394 MCI individuals from the Alzheimer’s Disease Neuroimaging Initiative, we studied whether an MCI diagnostic requirement of impairment on at least two episodic memory tests improves 3-year prediction of medial temporal lobe atrophy and progression to AD. Based on external age-adjusted norms for delayed free recall on the Rey Auditory Verbal Learning Test (AVLT), MCI participants were further classified as having normal (AVLT+, above −1 SD, *n* = 121) or impaired (AVLT -, −1 SD or below, *n* = 273) AVLT performance. CN, AVLT+, and AVLT- groups differed significantly on baseline brain (hippocampus, entorhinal cortex) and cerebrospinal fluid (amyloid, tau, p-tau) biomarkers, with the AVLT- group being most abnormal. The AVLT- group had significantly more medial temporal atrophy and a substantially higher AD progression rate than the AVLT+ group (51% vs. 16%, *p* < 0.001). The AVLT+ group had similar medial temporal trajectories compared to CN individuals. Results were similar even when restricted to individuals with above average (based on the CN group mean) baseline medial temporal volume/thickness. Requiring impairment on at least two memory tests for MCI diagnosis can markedly improve prediction of medial temporal atrophy and conversion to AD, even in the absence of baseline medial temporal atrophy. This modification constitutes a practical and cost-effective approach for clinical and research settings.

**Electronic supplementary material:**

The online version of this article (10.1007/s11682-018-0019-6) contains supplementary material, which is available to authorized users.

## Introduction

The pathological process in Alzheimer’s disease (AD) begins long before the onset of dementia (Braak et al. 2011; Jack et al. 2010) making early detection a primary concern. To aid in early detection, mild cognitive impairment (MCI) has been introduced as a prodromal stage of AD. However, MCI can arise from causes other than AD (Albert et al. 2011; Sperling et al. 2011). Improvement in MCI diagnosis is needed to ensure that those with MCI are actually at increased risk of progressing to AD.

Although individuals with MCI are at elevated risk for developing dementia, there is substantial variation in progression rates across studies (Langa and Levine 2014). Amyloid and tau biomarkers are used to support a diagnosis of AD in research studies, and the National Institute on Aging-Alzheimer’s Association (NIA-AA) framework also recommends inclusion of these biomarkers for earlier identification of individuals in preclinical or prodromal stages of the disease (Jack et al. 2018). However, evidence suggests that cognitive deficits may be able to predict progression to AD at an even earlier stage (Edmonds et al. 2015; Gomar et al. 2011; Jedynak et al. 2012, 2015).

The core clinical criteria of the NIA-AA definition of MCI refer to impairment in one or more cognitive domains (Albert et al. 2011); however no definition of cognitive impairment is provided. Age- and education-adjusted scores falling 1 or 1.5 standard deviations below that expected for age and education level may indicate MCI but these are considered as guidelines rather than diagnostic cut-offs. Importantly, there is no recommendation about the number of tests that must show impairment within a domain.

The Alzheimer’s Disease Neuroimaging Initiative (ADNI) criteria for amnestic MCI include a score lower than that expected for education level on delayed recall of the Wechsler Memory Scale (WMS) story A (Petersen et al. 2010). Prior neuropsychological studies indicate that reliance on a single measure is problematic because impaired scores on at least one measure are common in neurologically normal adults given a large battery of tests (Heaton et al. 2004). Memory is also phenotypically and genetically complex. Different memory tests are not all influenced by the same genes and do not manifest the same degree of age-related change (Kremen et al. 2014b; Panizzon et al. 2011; Papassotiropoulos and de Quervain 2011). Relying on a single neuropsychological test to define impairment is thus likely to be sub-optimal. Because gauging memory impairment is easier and less expensive than assessing cerebrospinal fluid (CSF) or neuroimaging biomarkers, it would be advantageous if the simple addition of an extra neuropsychological test could aid in early diagnosis and prognosis of MCI.

Cognitive deficits are, by definition, more subtle in MCI than in dementia. As such, more extensive testing is important for adequate sensitivity (Kremen et al. 2014a). The Jak/Bondi approach, an actuarial-neuropsychological diagnosis of MCI, provides strong support for this notion (Bondi et al. 2014; Jak et al. 2009). Compared to the ADNI MCI diagnoses, when diagnosis was based on the Jak/Bondi approach, there was a smaller proportion reverting to normal, a higher proportion progressing to AD, a higher proportion with at least one *APOE*-ε4 allele, and higher proportions with abnormal CSF levels of Aβ and tau; thus, this approach appeared to improve identification of individuals with prodromal AD (Bondi et al. 2014; Jak et al. 2009).

Cognitive measures are strong predictors of progression from amnestic MCI to AD, sometimes even better than biomarkers (Apostolova et al. 2010; Chang et al. 2010; Ewers et al. 2012; Gomar et al. 2011, 2014; Heister et al. 2011; Landau et al. 2010; Moradi et al. 2016). In computational models of progression to AD, changes in delayed recall on the Rey Auditory Verbal Learning Test (AVLT)—a widely used list-learning test—occurred prior to other indicators (Jedynak et al. 2012, 2015). Such findings challenge the notion that cognitive deficits are always identified last in the progression to AD (Edmonds et al. 2015; Jack et al. 2010, 2013). Importantly, some ADNI MCI participants also performed well on the AVLT, indicating a logical inconsistency in the diagnosis of amnestic MCI that highlights the importance of employing more than one test. That is, can someone truly have memory impairment if they perform normally on the AVLT?

In the present study, we compared three groups of ADNI participants: cognitively normal (CN) individuals; amnestic MCI with normal AVLT performance (AVLT+); and amnestic MCI with impaired AVLT performance (AVLT-). The definition of normal and impaired AVLT delayed recall performance was based on the age-adjusted Mayo Older Americans Normative Studies (MOANS) (Steinberg et al. 2005). We examined validators of MCI diagnosis: baseline hippocampal volume and entorhinal cortex thickness; baseline CSF Aβ_1–42_, tau and phosphorylated tau (p-tau); change in hippocampal volume and entorhinal cortex volume over time; and progression to AD. We hypothesized that including just this one additional memory test would improve diagnostic precision and prediction, i.e., it would result in higher rates of progression to AD and greater medial temporal atrophy over time. We also tested whether this effect would be present even in those without evidence of medial temporal neurodegeneration. If so, it would constitute a labor- and cost-efficient improvement for the core clinical and research criteria for MCI.

## Materials and methods

### Participants

Data were obtained from the ADNI database (http://adni.loni.usc.edu) (Mueller et al. 2005; Petersen et al. 2010). The ADNI began in 2003 as a public-private partnership with Michael W. Weiner, M.D. as the principal investigator. Its primary goal has been to determine whether combinations of longitudinal neuroimaging, other biological markers, and clinical and neuropsychological assessments can measure the progression of MCI and early AD.

The present study included 624 participants with AVLT data: 394 who fulfilled ADNI criteria for MCI and 230 who were CN at baseline. CSF measures were available for 308–312 participants. Baseline brain measures were available for 569 participants. The number of participants in longitudinal brain analyses varied for each time point: 6 month [m] = 448; 12 m = 402; 18 m = 216; 24 m = 327; 36 m = 169.

### ADNI MCI diagnosis

Diagnosis of amnestic MCI was made according Petersen et al. criteria: objective memory impairment defined by education-adjusted scores ≥1.5 SDs below the normative mean on delayed recall of WMS Story A; subjective memory complaints; global Clinical Dementia Rating Scale score of 0.5; and Mini-Mental State Examination score ≥ 24 (Petersen et al. 2010).

### Demographics

Demographics included age, sex, education, and the American National Adult Reading Test (ANART) as a measure of premorbid cognitive ability. *APOE* genotype status was based on presence/absence of an ε4 allele.

### Rey auditory verbal learning test (AVLT)

The AVLT includes five learning trials of a 15-word list followed by an interference list, recall of the first list, and 20-min delayed recall of the first list. We used the age-specific norms from the MOANS (Steinberg et al. 2005). We further categorized those with MCI based on a cutoff of 1 SD below the mean on AVLT delayed recall: AVLT- (scaled score ≤ 7); and AVLT+ (scaled score ≥ 8). We used a more liberal threshold for defining AVLT impairment because, by definition, MCI participants were already ≥1.5 SDs below the normative mean on the WMS (Jak et al. 2009). In a secondary analysis, we also investigated progression to AD in scaled-score groups separately.

### Biomarkers

The ADNI Biomarker Core Laboratory at the University of Pennsylvania used standardized procedures to measure Aβ_1–42_, tau and p-tau_181p_ in CSF (Shaw 2008). Low CSF levels of Aβ_1–42_ are thought to reflect accumulation of amyloid in senile plaques in the brain (Zwan et al. 2016). Elevated CSF levels of tau and p-tau are thought to reflect neurofibrillary tangles (Zetterberg 2017). We used previously established cutoffs for these measures (Shaw et al. 2009). ADNI participants underwent brain magnetic resonance imaging with 1.5 T scanners. We examined two key Alzheimer’s-related medial temporal lobe regions of interest: bilateral hippocampal volume and entorhinal cortex thickness based on FreeSurfer 5.1 (Dale et al. 1999; Fischl et al. 1999, 2002). Change over time in these structures was quantified using Quarc (Holland et al. 2011, 2012).

### Statistical analysis

We first report prevalence rates, means, SDs, and χ^2^ and t-tests comparing CN and ADNI-defined MCI participants. Next, we report corresponding statistics comparing our AVLT+ and AVLT- MCI subgroups. We used linear regression models with the AVLT+ group as a reference in analyses of baseline differences in CSF biomarkers and brain measures. Figures contain raw values for the CSF and brain measures, but the *P*-values are based on models with age and sex as covariates in the CSF analyses, and age, sex and estimated intracranial volume as covariates in the neuroimaging analyses.

We used mixed models to investigate rate of change in hippocampal volume and entorhinal cortices thickness. Percent change from baseline was assessed at 6, 12, 18, 24 and 36 months; per the ADNI protocol, CNs were not tested at 18 months. Slopes for brain atrophy were estimated by including an interaction term between diagnostic group and visit month of follow-up.

Logistic regression models were used to compare the prevalence of AD for AVLT+ and AVLT- groups at each time point. Cox proportional hazard models with the Breslow method for ties were used to examine progression to AD in AVLT+ and AVLT- groups. We also examined conversion to AD separately in different AVLT scaled-score categories.

To test whether we could observe cognitive impairment in the absence of neurodegeneration, we compared subgroups of individuals who had no neurodegeneration at baseline. These analyses included only individuals whose hippocampal volume or entorhinal cortex thickness was greater than the CN group mean at baseline.

We considered a *P* value <.05 threshold for statistical significance. Analyses were performed using Stata version 13.

## Results

### Descriptive statistics

There were significantly (χ^2^ = 8.66, *P* < .01) more men in the MCI group (64%, 254/396) than in the CN group (52%, 120/230). CN participants were older (*P* < .05) and had higher ANART scores (*P* < .001) than those with MCI but educational level did not differ between these two groups (*P* = .14). Having an *APOE* ε4 allele was more common (χ^2^ = 42.52, *P* < .001) in participants with MCI (54%) than in the CN group (27%) (Table 1).

**Table 1:** Demographic and memory measures in cognitively normal individuals (CN) and those with amnestic mild cognitive impairment (MCI) according to the Alzheimer’s Disease Neuroimaging Initiative criteria, and in the two MCI subgroups classified according to performance on the Rey Auditory Verbal Learning Test (AVLT) delayed free recall

	CN (*n* = 230)		MCI (*n* = 394)			AVLT+ (*n* = 121)		AVLT- (*n* = 273)		
	M	SD	M	SD	t	M	SD	M	SD	t
Age	76.12	5.02	74.92	7.44	2.40*	76.77	7.19	74.14	7.36	3.29**
Education	16.03	2.85	15.67	3.04	1.48	16.02	2.84	15.51	3.13	1.52
ANART	40.28	9.13	36.33	9.90	5.02***	37.06	10.38	36.01	9.68	0.97
AVLT 1	5.17	1.66	4,19	1.53	7.43***	4.81	1.63	3.92	1.40	5.52***
AVLT 5	11.02	2.35	7.47	2.58	17.01***	9.76	2.55	6.45	1.85	12.88***
AVLT 1–5	43.35	9.13	30.64	8.97	16.85***	38.08	9.54	27.34	6.36	11.33***
AVLT delayed	7.42	3.70	2.81	3.26	15.49***	6.76	2.89	1.07	1.29	20.80***

*ANART* American National Adult Reading Test, *MCI* mild cognitive impairment diagnosis according to ADNI criteria; AVLT + = MCI individuals with normal performance in Rey Auditory Verbal Learning Test, defined as age adjusted score of better than −1 SD; AVLT - = MCI individuals with impaired performance in Rey Auditory Verbal Learning Test, defined as age adjusted score of −1 SD or below; AVLT 1 = number of correct words in AVLT trial 1; AVLT 5 = number of correct words in AVLT trial 5; AVLT 1–5 = number of correct words in AVLT trials 1–5; AVLT del = number of correct words in AVLT delayed free recall; Education indicate years of education. ANART indicate number of correctly pronounced words

*P < .05; **P < .01; ***P < .001

The AVLT- group (*n* = 273) was younger than the AVLT+ group (*n* = 121) (*P* < .01), but there were no differences in educational level (*P* = .13) or ANART performance (*P* = .34), and the sex ratios were similar (χ2 = 0.00, *P* = .999, 64% men in both groups, 176 men in the AVLT- and 78 men in the AVLT+ groups) (Table 1). Having an *APOE*-ε4 allele was significantly (χ2 = 14.04, *P* < .001) more common in AVLT- group (60%) than the AVLT+ group (40%). Not surprisingly, these groups also differed significantly on other AVLT measures (Table 1, Online Resource: Supplementary Fig. 1).

### Baseline CSF measures

**Fig. 1:**
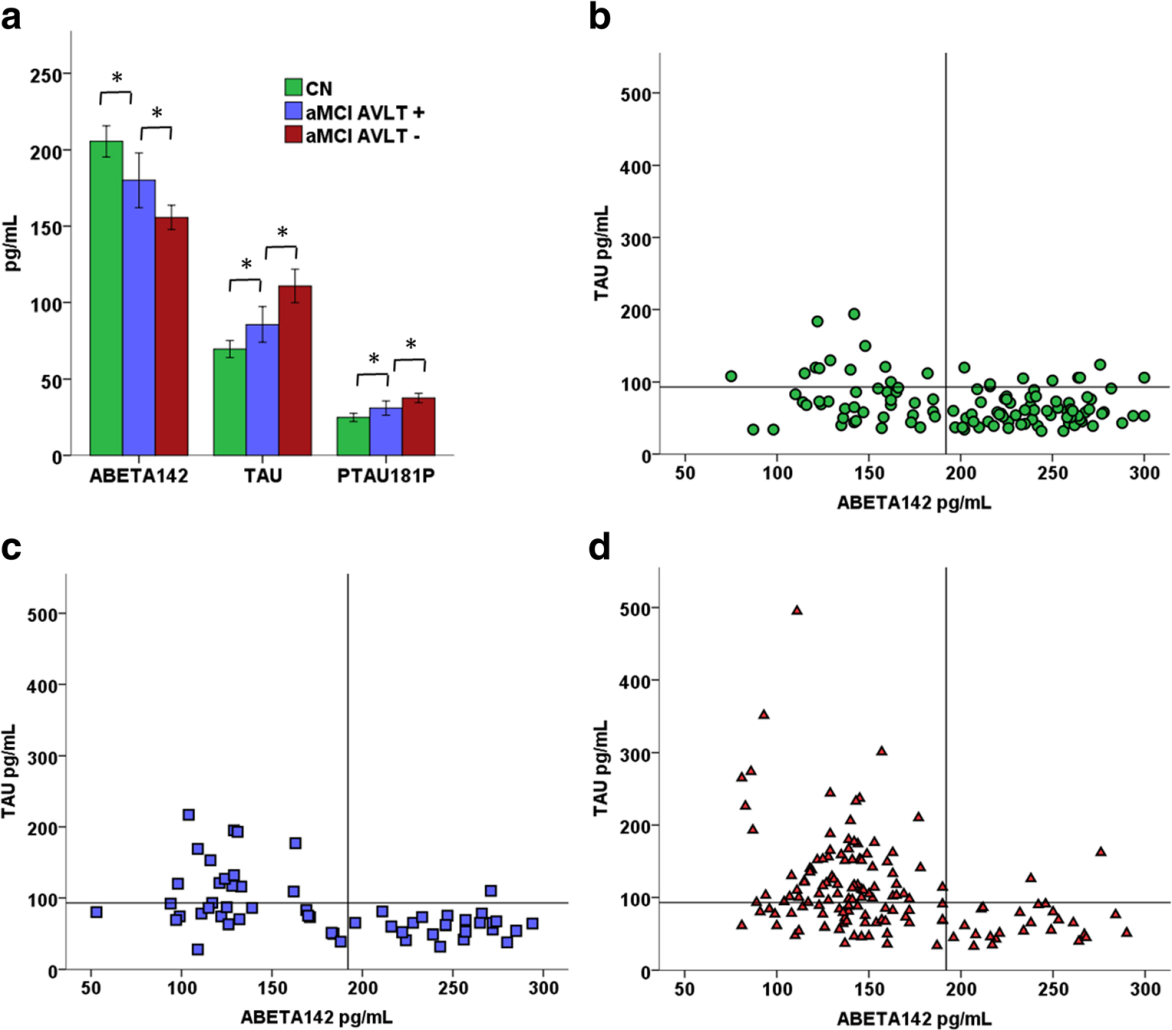
Baseline cerebrospinal fluid levels of β-amyloid (ABETA142), total tau (TAU) and phosphorylated tau (PTAU181). **a** Means with 95% confidence intervals in cognitively normal participants (CN) and in those with amnestic mild cognitive impairment either with good (aMCI AVLT+) or impaired (aMCI AVLT-) Auditory Verbal Learning Test performance. * = statistically significant (*p* < 0.05) difference between groups. **b** scatterplot of β-amyloid and total tau in CN group, **c** scatterplot of β-amyloid and total tau in the aMCI AVLT+ group, **d** scatterplot of β-amyloid and total tau in the aMCI AVLT- group, with cutoff values from Shaw et al. 2009, 65, 403–413 Annals of Neurology

The three groups differed on all CSF biomarkers (Table 2, Fig. 1a–d). Aβ_1–42_ level was significantly (*t* = 2.77, *P* = .006) higher in the CN group than the AVLT+ group, which in turn had significantly higher Aβ_1–42_ levels compared to AVLT– group (*t* = −3.11, *P* = .002). Both tau and p-tau_181p_ levels were significantly lower in the CN group (tau: *t* = −2.06, *P* = .040; p-tau_181p_: *t* = −2.16, *P* = .031) than the AVLT+ group, and in the AVLT+ group compared with the AVLT- group (tau: *t* = 3.37, *P* = .001; p-tau_181p_: *t* = 2.83, *P* = .005).

**Table 2:** Baseline cerebrospinal fluid (CSF) and brain biomarkers in cognitively normal individuals (CN) and two subgroups of amnestic mild cognitive impairment individuals classified according to performance on the Rey Auditory Verbal Learning Test (AVLT) delayed free recall. AVLT+ group is significantly different from CN and AVLT- groups in all biomarkers

	CN		AVLT+		AVLT-	
	M	SD	M	SD	M	SD
CSF Aβ_1–42_ (pg/ml)	205.59	55.09	181.39	65.81	155.58	47.17
CSF tau (pg/ml)	69.68	30.37	85.69	42.70	110.78	65.72
CSF p-tau_181p_ (pg/ml)	24.86	14.58	30.61	17.14	37.64	18.07
Hippocampal volume (mm^3^)	3631	440	3432	470	3159	522
Entorhinal cortical thickness (mm)	3.25	0.30	3.12	0.44	2.85	0.45

AVLT + = MCI individuals with normal performance in Rey Auditory Verbal Learning Test, defined as age adjusted score of better than −1 SD; AVLT - = MCI individuals with impaired performance in Rey Auditory Verbal Learning Test, defined as age adjusted score of −1 SD or below

The proportion of those with both abnormal Aβ_1–42_ (<192 pg/ml) and abnormal t-tau (>93 pg/ml) levels was significantly (*P* < .001) higher in AVLT- group (49.6%) than the AVLT+ group (23.6%). Also, the proportion of those with both Aβ_1–42_ and tau levels in the normal range was lower in the AVLT- group (17.3%) (Fig. 1d) compared to AVLT+ group (40.0%) (Fig. 1c). In CN participants, just over half (54.4%) had normal levels of both Aβ_1–42_ and total tau, whereas only 10.5% had abnormal levels of both (Fig. 1b).

### Baseline brain measures

CN participants had significantly greater hippocampal volume (*t* = 3.49, *P* = .001) and thicker entorhinal cortex (*t* = 2.85, *P* < .001) than the AVLT+ group (Table 2, Online Resource Supplementary Fig. 2). The AVLT- group had significantly smaller hippocampal volume (*t* = −4.86, *P* < .001) and thinner entorhinal cortex (*t* = −5.74, *P* < .001) than the AVLT+ group (Table 2, Online Resource Supplementary Fig. 2).

### Longitudinal brain measures

**Fig. 2:**
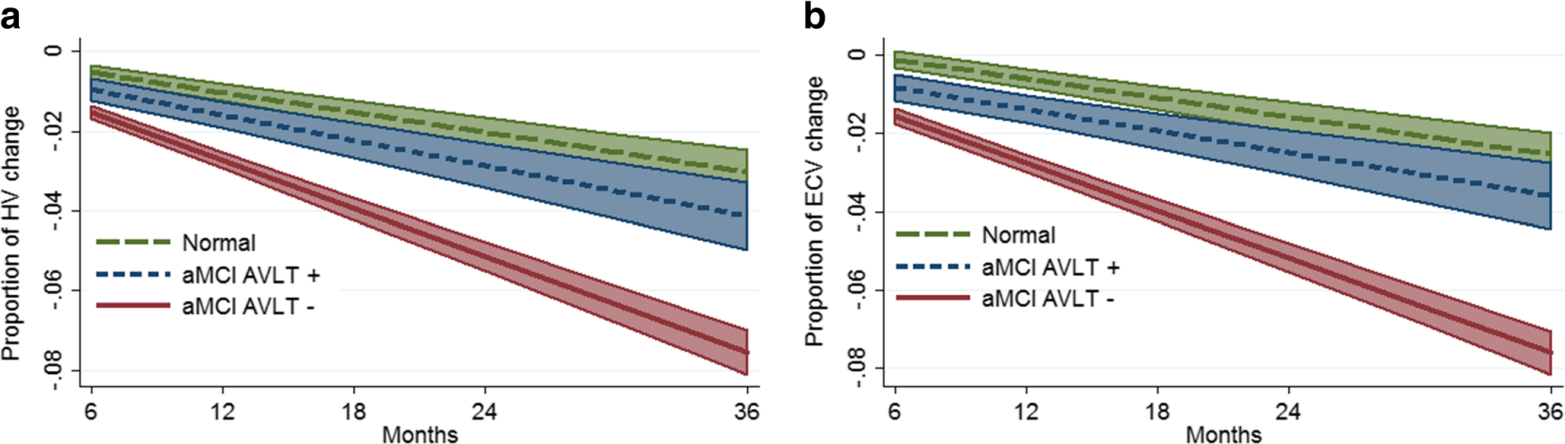
Volume change as a proportion of baseline size from 6 to 36 months in cognitively normal participants (CN) and in those with amnestic mild cognitive impairment with normal (aMCI AVLT+) or impaired (aMCI AVLT-) Auditory Verbal Learning Test performance for hippocampus (HV; panel **a**) and entorhinal cortex (ECV, panel **b**)

All groups had significant negative slopes for hippocampal volume (CN slope = −.0050 [95%CI: −.0059; −.0040]; AVLT+ slope = −.0064 [95%CI: −.0079; −.0048]; AVLT- slope = −.0120 [95%CI: −.0130; −.0110]) and entorhinal cortex volume (CN slope = −.0048 [95%CI: −.0058; −.0038]; AVLT+ slope = −.0055 [95%CI: −.0071; −.0040]; AVLT- slope = −.0121 [95%CI: −.0131; −.0111]) (Fig. 2a and b; Online Resource Supplementary Tables 1–2).

The AVLT- group had significantly steeper negative trajectories of hippocampal (z = −9.94, *P* < .0001, Fig. 2a) and entorhinal cortical volumes (z = −10.12, *P* < .0001, Fig. 2b) compared to CN participants. However, the slopes of both hippocampal (z = −1.48, *P* = .139, Fig. 2a) and entorhinal cortical volumes (z = −0.73, *P* = .464, Fig. 2b) did not differ between the CN and AVLT+ groups.

### Progression to AD

**Fig. 3:**
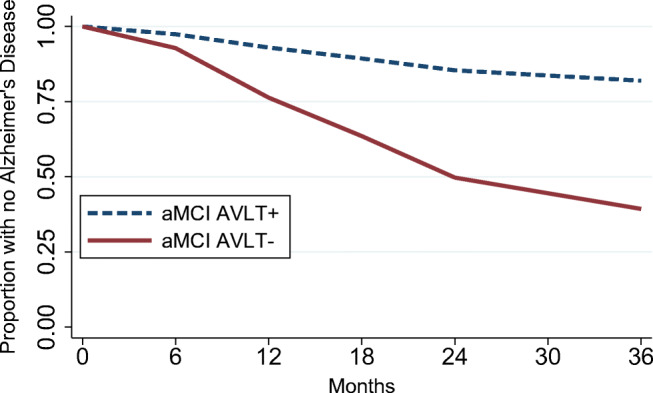
Kaplan-Meier survival estimates in individuals with amnestic mild cognitive impairment either with good (aMCI AVLT+) or impaired (aMCI AVLT-) Rey Auditory Verbal Learning Test performance

The AVLT- group had substantially higher risk than the AVLT+ group of progression to AD (HR = 4.39 [95%CI: 2.70; 7.13], z = 5.96, *P* < .001, Fig. 3). During the follow-up, 50.5% (138/273) of the AVLT- group met criteria for AD compared to only 15.7% (19/121) of the AVLT+ group. When we included *APOE* status as an additional covariate in the model, having *APOE* ε4 allele was associated with increased risk of progression to AD (HR = 1.81 [95%CI: 1.28; 2.55], z = 3.35, *P* < .001). However, the overall result changed little even after controlling for *APOE* status (HR = 4.02 [95%CI: 2.46; 6.57], z = 5.57, *P* < .001). Online Resource Supplementary Table 3 shows the prevalence of AD at each time point separately for conventional ADNI MCI criteria and for the AVLT+ and AVLT- groups.

Participants with AVLT scaled scores of 3–7 had similar risk of progression to AD compared to the reference group with the lowest score of 2 (*P*s > .05, Supplementary Fig. 3, Supplementary Table 4). Participants with scores of 8 or higher had significantly lower risk of progression to AD compared to those with a score of 2 (*P*s < .05, Online Resource Supplementary Fig. 3 & Supplementary Table 4).

### Subgroup analysis of individuals without baseline neurodegeneration

The brain trajectory results were similar when we included only those with hippocampal volume or entorhinal cortical thickness that was equal or greater than the CN group mean: hippocampal volume ≥ 3631 mm^3^; entorhinal cortical thickness ≥ 3.25 mm. In these analyses, the AVLT- group did not differ from CN and AVLT+ groups in baseline hippocampal volume or entorhinal cortical thickness (all *P*s = .177–.421). Nevertheless, the AVLT- group had a significantly steeper negative trajectory of hippocampal volume (z = −261, *P* = .009) and entorhinal cortex (z = −2.50, *P* = .012) compared to CN participants. In contrast, the slopes for both hippocampal volume (z = −0.41, *P* = .680) and entorhinal cortex (z = −0.11, *P* = .912) change did not differ between the CN and AVLT+ groups.

In those with above average hippocampal volume, the AVLT- group (35.7%, 15/42) still had a significantly higher progression rate than the AVLT+ group (7.9%, 3/38) (HR = 5.27 [95%CI: 1.41; 19.67], z = 2.47, *P* = .013). Similarly, the AVLT- group (31.9%, 15/47) had significantly higher risk of progression to AD than AVLT+ group (15.9%, 7/44) when including only those with above average baseline entorhinal cortical thickness (HR = 2.63 [95%CI: 1.06; 6.55], z = 2.08, *P* = .037). The results were similar in both cases even after controlling for Aβ_1–42_ (27.3% [6/22] vs. 6.3% [1/16]; HR = 3.25 [95%CI: 0.39; 27.06], z = 1.09, *P* = .275) for hippocampal volume and (31.8% [7/22] vs. 18.2% [4/22]; HR = 1.73 [95%CI: 0.46; 6.57], z = 0.81, *P* = .418) for entorhinal cortex. Despite the similar results, these differences were not significant due to the reduced sample size for participants with Aβ_1–42_ or hippocampal/entorhinal cortex data. In individuals with both above average hippocampal volume and entorhinal cortex thickness, more AVLT- individuals (26.3%, 5/19) than AVLT+ individuals (11.1%, 3/27) progressed to AD, but this difference was only at trend level in this even smaller subgroup (HR = 2.71 [95%CI: 0.63; 11.59], z = 1.34, *P* = .179).

## Discussion

A body of evidence supports the idea that more extensive assessment with more than one measure in each cognitive domain improves diagnostic accuracy (Bondi et al. 2014; Edmonds et al. 2016; Jak et al. 2009). Several studies have used the AVLT along with CSF and brain biomarkers as predictors of progression from ADNI-diagnosed MCI to AD (Apostolova et al. 2010; Chang et al. 2010; Ewers et al. 2012; Gomar et al. 2011, 2014; Heister et al. 2011; Landau et al. 2010; Moradi et al. 2016). In these studies, the AVLT was treated as an external predictor despite the fact that AVLT scores sometimes conflicted with the core clinical criteria for diagnosis. Here we examined the impact of simply adding this one additional episodic memory measure to the diagnostic criteria, thereby creating AVLT+ and AVLT- subgroups.

More AVLT- participants than AVLT+ participants had an *APOE* ε4 allele and twice as many AVLT- participants as AVLT+ participants had baseline levels of CSF beta amyloid and tau consistent with AD (Shaw et al. 2009). AVLT- participants also had significantly smaller baseline hippocampal volume and entorhinal cortical thickness compared to AVLT+ participants and greater rates of atrophy over time. Most importantly, over three times as many AVLT- participants progressed to AD during the 36-month follow-up compared with AVLT+ participants. Taken together, these results strongly support the validity of our MCI diagnostic modification, leading us to recommend that the core clinical criteria defining amnestic MCI should incorporate the criterion of impaired performance on at least two memory measures.

In keeping with the NIA-AA recommendations (Albert et al. 2011), it is also essential that the degree of cognitive impairment be abnormal for one’s age. Two studies defined single AVLT impairment cutpoints derived by comparing CN and AD ADNI participants (Heister et al. 2011; Landau et al. 2010). The goal of these studies was not to modify the MCI diagnostic criteria, and their uniform cutpoint would not be optimal for defining MCI because there are substantial age differences on AVLT performance. For example, an average score for 85-year olds is 1 SD below the mean for 60-year olds (Steinberg et al. 2005). Also, the original ADNI MCI criteria used education-adjusted scores of WMS story recall, but scores adjusted for both age and education are likely to further improve MCI diagnosis.

One study of ADNI participants categorized individuals with MCI based on the number of impaired tests and found that this criterion worked better than the original ADNI MCI classification or the Jak/Bondi actuarial approach in predicting progression from MCI to AD (Oltra-Cucarella et al. 2018). This study used the average number of low scores in the worst performing 10% of ADNI CN participants as the basis for diagnosing MCI. Low scores were defined as performance of ≥1.5 SD below the mean of the CN ADNI participants. Out of 9 scores from 6 tests, the lowest 10% of CN participants had ≥3 low scores. The highest progression rate (43%) to AD in a 3-year period was in those with single domain amnestic MCI (i.e., individuals who were ≥ 1.5 SD below the mean in Logical Memory delayed recall, AVLT delayed recall and AVLT recognition) (Oltra-Cucarella et al. 2018). This rate was higher than the progression rate of 33% for multiple-domain amnestic MCI, probably because one could meet criteria for multiple-domain amnestic MCI with only one or two impaired memory scores but a single-domain diagnosis would require impairment on all three. This approach may not be easily transferable into clinical use for two reasons. First, the cutoff for impairment was based on the distribution of scores in the ADNI sample rather than external norms. Second, the criterion of three impaired scores in the lowest 10% subgroup came from a set of 9 scores, but the number of impaired tests in the lowest 10% will vary as a function of how many are administered. Also, caution is warranted when counting certain scores from the same test. For example, almost all individuals with impaired AVLT recognition will have impaired AVLT recall. It is probably optimal to use recall measures from two different tests, particularly for diagnosing MCI when recognition deficits will be much less common than in AD. Our approach simply added a second memory recall test, and it resulted in a higher 3-year progression rate of 51%.

With 15.7% of the AVLT+ group progressing to AD, it might be that some people with only one impaired memory measure are in earlier stages of MCI. This may raise concern about false negatives. Our results are consistent with prior neuropsychological studies indicating that threshold yields too many false positives (Heaton et al. 2004; Palmer et al. 1998), but direct comparisons of ADNI diagnoses with Jak/Bondi diagnoses have also been consistent with ADNI diagnoses resulting in more false negatives (Bondi et al. 2014; Edmonds et al. 2016). Indeed, 8% of the CN group had AVLT scores >1.5 SDs below normative means. If diagnosis requires only one impaired memory measure, this could indicate up to 8% false negatives. We also observed a significantly higher proportion of *APOE* ε4 allele carriers in those with two impaired tests. However, the group differences in progression to AD held up even after controlling for *APOE* status. This suggests that the AVLT- group may be at greater genetic risk for AD, but it also indicates that the group differences were not simply driven by *APOE*.

The AVLT- group had the most baseline CSF and brain biomarker abnormalities. According to the amyloid/tau/neurodegeneration (A/T/(N)) framework (Jack et al. 2018, memory impairment occurs subsequent to A/T/(N). However, when we included only individuals with above average hippocampal volume, entorhinal cortex thickness, or both, relative to the CN group mean—i.e., those with no medial temporal neurodegeneration—the AVLT- group still had significantly steeper trajectories of brain atrophy and progression rates than the AVLT+ group. Although power was limited, the magnitude of increased risk in the AVLT- group was similar even after controlling for Aβ, suggesting that the differences were not driven simply by amyloidosis.

The representativeness of ADNI is a limitation of our study (Petersen et al. 2010). Over 90% of ADNI participants are white and both CN individuals and those with MCI had a mean education of 16 years, corresponding to four-year university degree. In contrast, U.S. census data indicate that only about 10% of people with birth years comparable to that of ADNI participants have a college education (Ryan and Bauman 2016). In line with the high educational level, ADNI participants have high estimated premorbid IQ levels, more than 1 SD above the population mean (Petersen et al. 2010). Additionally, ADNI excluded individuals who were likely to suffer from other diseases that can affect cognition. Thus this approach requires validation in a more representative sample.

In sum, we showed that simply employing two recall tests, rather than one, substantially improved the validity of MCI diagnoses by reducing false positives with respect to prediction of medial temporal atrophy and progression to AD over a 3-year period. We showed essentially the same pattern even in individuals with above average baseline medial temporal volumes while controlling for biomarker levels. Although there is as yet no definitive determination as to just how extensive a test battery needs to be for optimizing the core clinical criteria for MCI, we recommend that requiring impairment on more than one recall memory test should be a criterion for the diagnosis of amnestic MCI. These findings are consistent with the view that cognitive impairment may not always come after biomarker and brain abnormalities in the progression to AD. Of course, assessing biomarkers and brain structures is still of great importance, but it may be that current detection thresholds do not always identify the earliest signs of biomarker or brain abnormalities. Moreover, on a practical level for clinical practice or screening for clinical trials, neuropsychological testing is low-cost and non-invasive in comparison to neuroimaging or CSF or PET biomarker assays.

## Electronic supplementary material

ESM 1 (DOCX 278 kb)
