# Supplementary material for: Modifying the minimum criteria for diagnosing amnestic MCI to improve prediction of brain atrophy and progression to Alzheimer’s disease
Source: Brain Imaging Behav. 2018 Dec 4;14(3):787–96. doi: 10.1007/s11682-018-0019-6 (PMC7275013; doi:10.1007/s11682-018-0019-6)
Supplement: Supplementary file 1 — (DOCX 278 kb) [file 11682_2018_19_MOESM1_ESM.docx]

**Modifying the minimum criteria for diagnosing amnestic MCI to improve prediction of brain atrophy and progression to Alzheimer’s Disease**

Eero Vuoksimaa, Linda K. McEvoy, Dominic Holland, Carol E. Franz, & William S. Kremen for the Alzheimer’s Disease Neuroimaging Initiative

**Supplementary material**

Supplemetary Tables: 4

Supplementary Figures: 3

**Supplementary Table 1. Hippocampal volume change (multiply by 100 to get % change) from baseline at each time point for cognitively normal individuals (CN) and two subgroups of amnestic mild cognitive impairment individuals classified according to performance on the Rey Auditory Verbal Learning Test (AVLT) delayed free recall.**

| Group | 6 M | 12 M | 18 M | 24 M | 36 M |
| --- | --- | --- | --- | --- | --- |
| CN (N) | 176 | 162 | 0 | 138 | 86 |
| M | -.0056906 | -.0108096 | . | -.0188202 | -.0325581 |
| SD | .0101939 | .0123178 | . | .0167561 | .0283227 |
|  |  |  |  |  |  |
| aMCI AVLT + (N) | 73 | 67 | 63 | 52 | 27 |
| M | -.0108504 | -.0158486 | -.0223754 | -.024095 | -.0453066 |
| SD | .013509 | .0196662 | .0224005 | .0209255 | .0328417 |
|  |  |  |  |  |  |
| aMCI AVLT - (N) | 199 | 173 | 153 | 137 | 56 |
| M | -.0153192 | -.0266371 | -.0398916 | -.0515802 | -.0782415 |
| SD | .0151535 | .0202029 | .0288403 | .0339644 | .0399541 |
|  |  |  |  |  |  |
| Total (N) | 448 | 402 | 216 | 327 | 169 |
| M | -.0108083 | -.0184607 | -.0347827 | -.0333841 | -.0497325 |
| SD | .0138262 | .0187956 | .028221 | .0301734 | .038993 |

Note. AVLT + = MCI individuals with normal performance in Rey Auditory Verbal Learning Test, defined as age adjusted score of better than -1 SD ; AVLT - = MCI individuals with impaired performance in Rey Auditory Verbal Learning Test, defined as age adjusted score of -1 SD or below.

**Supplementary Table 2. Entorhinal cortical volume change (multiply by 100 to get % change) from baseline at each time point for cognitively normal individuals (CN) and two subgroups of amnestic mild cognitive impairment individuals classified according to performance on the Rey Auditory Verbal Learning Test (AVLT) delayed free recall.**

| Group | 6 M | 12 M | 18 M | 24 M | 36 M |
| --- | --- | --- | --- | --- | --- |
| CN (N) | 176 | 162 | 0 | 138 | 86 |
| M | -.0013013 | -.0076073 | . | -.0157174 | -.0254372 |
| SD | .017568 | .016059 | . | .0195638 | .0274109 |
|  |  |  |  |  |  |
| aMCI AVLT + (N) | 73 | 67 | 63 | 52 | 27 |
| M | -.0091044 | -.013794 | -.0214472 | -.0222899 | -.0362176 |
| SD | .0133517 | .0183041 | .0247991 | .0251181 | .0406149 |
|  |  |  |  |  |  |
| aMCI AVLT - (N) | 199 | 173 | 153 | 137 | 56 |
| M | -.0158061 | -.0269268 | -.0384488 | -.0519429 | -.0803386 |
| SD | .0139933 | .0181677 | .0228529 | .0303616 | .0399392 |
|  |  |  |  |  |  |
| Total (N) | 448 | 402 | 216 | 327 | 169 |
| M | -.0090158 | -.0169525 | -.03349 | -.0319396 | -.0453517 |
| SD | .0167404 | .0194969 | .0246285 | .0306353 | .042215 |

Note. AVLT + = MCI individuals with normal performance in Rey Auditory Verbal Learning Test, defined as age adjusted score of better than -1 SD ; AVLT - = MCI individuals with impaired performance in Rey Auditory Verbal Learning Test, defined as age adjusted score of -1 SD or below.

**Supplementary Table 3. Number of individuals with amnestic mild cognitive impairment (MCI) according to conventional Petersen criteria used in the Alzheimer’s Disease Neuroimaging Initiative, and in two MCI groups according to their performance in the Rey Auditory Verbal Learning Test (AVLT) delayed free recall. Odds ratios (OR) with 95% confidence intervals (CI) for the risk of Alzheimer’s disease (AD) for those who had normal AVLT performance at baseline (AVLT+) compared to those with impaired (≤ -1 SD) AVLT performance (AVLT-).**

|  | Conventional | AVLT based | | AVLT+ vs. AVLT- |
| --- | --- | --- | --- | --- |
|  | MCI | AVLT + | AVLT – | OR (95% CI) |
| 6 months | 377 | 113 | 264 |  |
| AD | 5.8% (22) | 2.7% (3) | 7.2% (19) | 2.64 (.76; 9.17) |
| 12 months | 355 | 110 | 245 |  |
| AD | 18.0% (64) | 5.5% (6) | 23.7% (58) | 5.38 (2.23; 12.97)* |
| 18 months | 319 | 100 | 219 |  |
| AD | 27.6% (88) | 10.0% (10) | 35.6% (78) | 5.99 (2.87; 12.50)* |
| 24 months | 295 | 96 | 199 |  |
| AD | 37.6% (111) | 13.5% (13) | 49.2% (98) | 6.90 (3.54; 13.47)* |
| 36 months | 219 | 74 | 145 |  |
| AD | 42.9% (94) | 16.2% (12) | 56.6% (82) | 7.89 (3.78; 16.45)* |

note. * = p <.001; OR’s based on logistic models with age and sex as covariates for each time point separately.

**Supplementary Figure 1. Distributions of A) Logical Memory immediate recall, B) Logical Memory delayed free recall, C) Auditory Verbal Learning Test (AVLT) total words in trials 1 – 5, and D) AVLT delayed free recall raw scores in those with mild cognitive impairment according to standard ADNI criteria. All measures indicate the number of words recalled.**

**
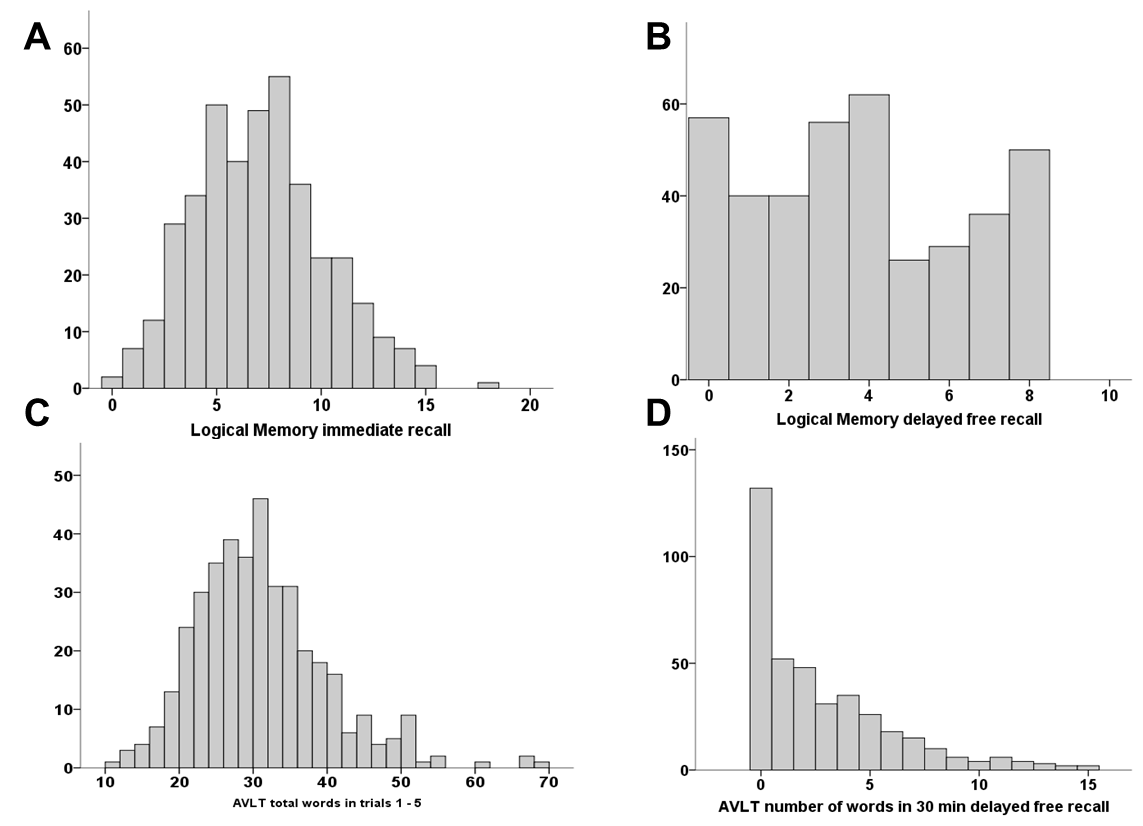
**

**Supplementary Figure 2. Baseline brain measures. Means and 95% confidence intervals of A) hippocampal volume (mm^3^), and B) entorhinal cortical thickness (mm) in cognitively normal participants (CN) and in those with amnestic mild cognitive impairment either with good (aMCI AVLT+) or impaired (aMCI AVLT-) Auditory Verbal Learning Test performance**


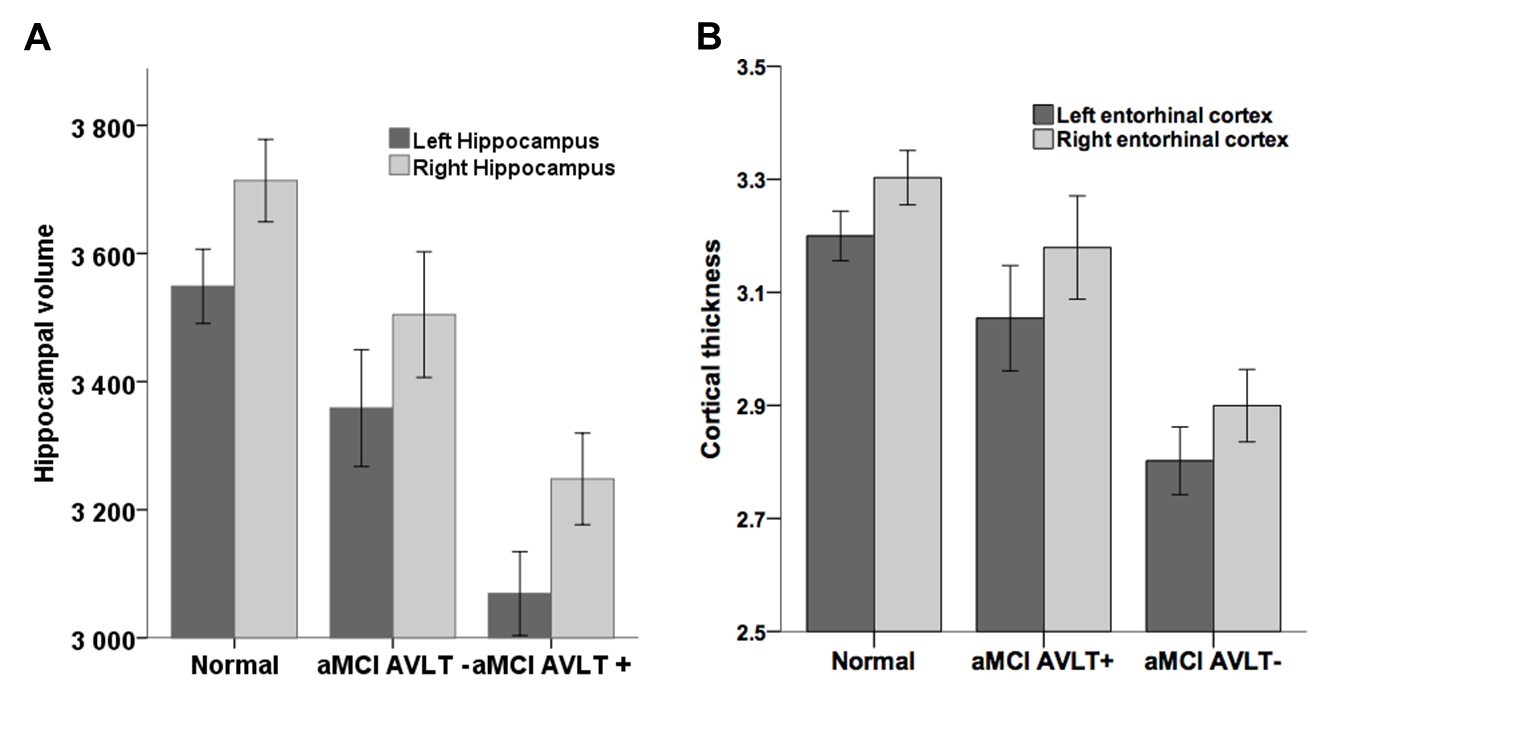


**Supplementary Figure 3. Kaplan-Meier survival estimates in individuals with amnestic mild cognitive impairment as a function of the age adjusted scaled scores of Rey Auditory Verbal Learning Test delayed free recall performance. Scores of ≥8 (dashed lines) indicate good performance (AVLT+) and scores of ≤7 (solid lines) indicated impaired performance (AVLT-).**

**Supplementary Table 4. Hazard ratios (HR) for individual scaled score groups (95% confidence intervals [CI’s] in parentheses, adjusted for age and sex). Reference group is scaled score of 2. Groups 12 – 18 are combined because these groups each had less than 10 individuals.**

| Scaled score | HR (95% CI’s) | p |
| --- | --- | --- |
| 3 | 1.20 (.52; 2.75) | 0.666 |
| 4 | .94 (.39; 2.27) | 0.883 |
| 5 | .86 (.38; 1.95) | 0.711 |
| 6 | .73 (.31; 1.72) | 0.470 |
| 7 | .53 (.21; 1.32) | 0.175 |
| 8 | .23 (.06; .82) | 0.023 |
| 9 | .27 (.09; .78) | 0.016 |
| 10 | .21 (.05; .80) | 0.023 |
| 11 | .06 (.01; .50) | 0.010 |
| 12-18 | .10 (.02; .40) | 0.001 |
